# Plasmin Promotes Keratinocyte Migration and Phagocytic-killing Accompanied by Suppression of Cell Proliferation which may Facilitate Re-epithelialization of Wound Beds

**DOI:** 10.1080/17402520400001710

**Published:** 2004

**Authors:** Imre Szabo, Miklos Simon, Janos Hunyadi

**Affiliations:** 1 Department of Dermatology Medical School of Debrecen Debrecen Hungary; 2 Department of Dermatology School of Medicine University of Erlangen Erlangen Germany

## Abstract

Abstract Keratinocytes were shown to induce the activation of plasminogen activator resulting in the formation of plasmin and the initiation of proteolysis *in vitro*. Activation of surface bound plasminogen may localize protease activity in the pericellular microenvironment and play a role in inducing both a conformational change and cell locomotion. Plasmin, however, can induce non-proteolytic effects on certain cell functions in a variety of cell lineages. In the present study we examined the effects of plasmin on keratinocytes with a focus on its role in the process of re-epithelialization, which included studies of cell migration, phagocytic-killing and cell proliferation. Migration of freshly isolated human epidermal keratinocytes was analyzed utilizing the agarose gel assay in the presence of 10% human serum. Plasmin at the concentration of 25 U/l induced a 160% increase in the chemotactic migration of keratinocytes that was completely blocked by the plasmin inhibitor α_2_-antiplasmin (Serpin). In the absence of serum, plasmin also induced a reversible chemotactic migration of HaCaT keratinocytes as determined utilizing the microchemotaxis assay. Dose-response analysis showed a bi-phasic effect of plasmin with a maximum increase of 52% in keratinocyte chemotaxis at a concentration of 25 U/l. HaCaT cells on the other hand, showed no detectable *in vitro* chemokinesis by plasmin. Phagocytic-killing of Candida albicans by freshly isolated epidermal keratinocytes was enhanced in the presence of 25 U/l plasmin which was also reversible by the addition of Serpin. Spontaneous proliferation of HaCaT keratinocytes as determined by ^3^H-Thymidine uptake on the other hand, was reduced by 47 and 13% in cultures with 25 U/l plasmin for 24 and 48 h respectively, in a Serpin reversible manner. These data suggest that plasmin-induced chemotactic migration of epidermal keratinocytes is accompanied by enhanced phagocytic-killing coupled with suppression of proliferation of these cells which may facilitate re-epithelialization following skin injury.


*Clinical & Developmental Immunology*, September/December 2004, Vol. 11 (3/4), pp. 233–240

Taylor & Francis healthsciences

### Plasmin Promotes Keratinocyte Migration and Phagocytic-killing Accompanied by Suppression of Cell Proliferation which may Facilitate Re-epithelialization of Wound Beds

IMRE SZABO[a], MIKLOS SIMON Jr.[b] and JANOS HUNYADI[a,*]

[a]*Department of Dermatology, Medical School of Debrecen, Debrecen, Hungary;* [b]*Department of Dermatology, School of Medicine, University of Erlangen, Erlangen, Germany*

Keratinocytes were shown to induce the activation of plasminogen activator resulting in the formation of plasmin and the initiation of proteolysis *in vitro*. Activation of surface bound plasminogen may localize protease activity in the pericellular microenvironment and play a role in inducing both a conformational change and cell locomotion. Plasmin, however, can induce non-proteolytic effects on certain cell functions in a variety of cell lineages. In the present study we examined the effects of plasmin on keratinocytes with a focus on its role in the process of re-epithelialization, which included studies of cell migration, phagocytic-killing and cell proliferation. Migration of freshly isolated human epidermal keratinocytes was analyzed utilizing the agarose gel assay in the presence of 10% human serum. Plasmin at the concentration of 25 U/l induced a 160% increase in the chemotactic migration of keratinocytes that was completely blocked by the plasmin inhibitor $\alpha_2$-antiplasmin (Serpin). In the absence of serum, plasmin also induced a reversible chemotactic migration of HaCaT keratinocytes as determined utilizing the microchemotaxis assay. Dose-response analysis showed a bi-phasic effect of plasmin with a maximum increase of 52% in keratinocyte chemotaxis at a concentration of 25 U/l. HaCaT cells on the other hand, showed no detectable *in vitro* chemokinesis by plasmin. Phagocytic-killing of *Candida albicans* by freshly isolated epidermal keratinocytes was enhanced in the presence of 25 U/l plasmin which was also reversible by the addition of Serpin. Spontaneous proliferation of HaCaT keratinocytes as determined by $^3$H-Thymidine uptake on the other hand, was reduced by 47 and 13% in cultures with 25 U/l plasmin for 24 and 48 h respectively, in a Serpin reversible manner. These data suggest that plasmin-induced chemotactic migration of epidermal keratinocytes is accompanied by enhanced phagocytic-killing coupled with suppression of proliferation of these cells which may facilitate re-epithelialization following skin injury.

*Keywords*: Keratinocyte; Migration; Phagocytic killing; Plasmin; Proliferation; Serpin

*Abbreviations*: HEK, human epidermal keratinocyte; PA, plasminogen activator; tPA, tissue type plasminogen activator; uPA, urokinase type plasminogen activator; R10, RPMI 1640+10% FBS; Serpin, serine protease inhibitor $\alpha_2$-antiplasmin

### INTRODUCTION

The plasminogen/plasmin system has been found to play a modulatory role in many physiological and pathophysiological processes of various cell lineages (Plow and Miles, 1990; Lijnen, 1996). Plasmin converted from the proenzyme plasminogen induces proteolysis of the pericellular glycoproteins resulting in disruption of cell–cell and cell–extracellular matrix adhesions (Reinartz *et al.*, 1993; Geer and Andreadis, 2003). Whereas plasminogen from serum or interstitial fluid can bind to the cell surface, there is also evidence for the intracellular synthesis of plasminogen, which can localize to the cell surface (Isseroff and Rifkin, 1983; Reinartz *et al.*, 1993; Venning *et al.*, 1993; Reinartz *et al.*, 1995). Plasminogen–plasmin conversion can be facilitated by the membrane-bound urokinase type (uPA) or tissue type (tPA) plasminogen activators (PA) expressed by activated

*Corresponding author. Address: Department of Dermatology, University of Debrecen, Medical and Health Sciences Center, Nagyerdei Blvd., 98, Debrecen H-4012, Hungary. E-mail: hunyadi@jaguar.unideb.hu

ISSN 1740-2522 print/ISSN 1740-2530 online © 2004 Taylor & Francis Ltd
DOI: 10.1080/17402520400001710

or even normal cells. Activation of membrane bound plasminogen may localize protease activity to the pericellular micro-environment (Blasi, 1993; Boxman *et al.*, 1995; Bechtel *et al.*, 1996; Chen and Jensen, 1996; Rox *et al.*, 1996). Plasmin activation has been reported to lead to an increase in cell mobility or spreading through enzymatic release of cell contacts, and play a role in certain physiological and pathological processes including wound healing, tumor cell invasion or embryonic development (Grondahl-Hansen *et al.*, 1988; Stephens *et al.*, 1989; Baird *et al.*, 1990; Del Rosso *et al.*, 1990; Meissauer *et al.*, 1992; Blasi, 1993; Reinartz *et al.*, 1994; Bechtel *et al.*, 1996).

Epidermal keratinocytes, especially basal and supra-basal cells have been shown to induce plasminogen activator activity (both uPA and tPA) in cell cultures and also during wound healing (re-epithelialization process) *in vivo* (Grondahl-Hansen *et al.*, 1988; Hashimoto *et al.*, 1988; McNeill and Jensen, 1990; Reinartz *et al.*, 1994; Ando and Jensen, 1996; Chen and Jensen, 1996; Reinartz *et al.*, 1996; Rox *et al.*, 1996; Xu *et al.*, 1997). Keratinocytes located at the leading edge of epithelial sheets express strong uPA activity during wound healing that is believed to contribute to keratinocyte migration and also to localize plasmin activation on the surface of migrating cells (Reinartz *et al.*, 1994). In spite of considerable experimental data, the effect of plasmin on keratinocytes is not completely understood. Therefore, we studied the effect of plasmin on keratinocyte function with a focus on those functions that have a major impact on the re-epithelialization process, including cell migration, phagocytosis and proliferation.

Our results show that plasmin induces chemotactic migration and enhanced phagocytic-killing activity of keratinocytes, but inhibits cell proliferation *in vitro*, suggesting an important regulatory role of plasmin in the re-epithelialization process *in vivo*.

### MATERIALS AND METHODS

### Culture Medium

RPMI-1640 medium supplemented with 2 mM L-glutamine (GibcoBRL, Grand Island, NY, USA), 10% heat inactivated fetal bovine serum (GibcoBRL) and 10 μl/ml antibiotic antimycotic solution (100 ×; Sigma, St Luis, MO, USA) was used for cell culture (R10) throughout the studies.

### Keratinocyte Cultures

Primary epidermal keratinocytes were freshly isolated from human skin utilizing standard methods (Rheinwald and Green, 1975) and were immediately used for experiments. Spontaneously immortalized human keratinocytes, HaCaT cells, were a kind gift of Professor N.E. Fusenig (University of Heidelberg, Germany). HaCaT keratinocytes were continuously cultured in R10 at 37°C in a 5% $CO_2$ atmosphere and cells that reached the pre-sheet culture stage were used for all experiments. For selected experiments keratinocytes were detached by the addition of 0.05% Trypsin–EDTA (Sigma), and the cell number was adjusted to $1 \times 10^6$/ml in R10. The viability of cultured and freshly isolated cells was monitored by the Trypan blue dye exclusion test, and cells with viability $> 95\%$ were used for experiments.

### Cell Migration Assays

#### *Agarose Gel Assay*

Keratinocyte migration in the presence of human serum was studied by the method of Nelson *et al.* (1975). Briefly, $2.5 \times 10^5$ freshly isolated human keratinocytes were placed in the middle wells of an agarose gel prepared with 10% heat inactivated AB serum (see Fig. 1). The central well was filled with medium (R10) and the outer wells with an appropriate chemoattractant. The outer wells were positioned 3 mm from the keratinocyte-containing middle wells, and each well was 3 mm in diameter. The plate was incubated for 24–48 h at 37°C in a 5% $CO_2$ incubator after which the spontaneous (toward the medium) and chemotactic migration of keratinocytes were evaluated by measuring the migration distance with the help of an inverted light microscope at low magnification. Chemotaxis was induced by 25 U/l Plasmin (Sigma) and was also determined in the presence of the plasmin inhibitor $\alpha_2$-antiplasmin (Serpin; Sigma) (Hall *et al.*, 1991) at the concentration of 25 U/l. The migration index was expressed as the ratio between

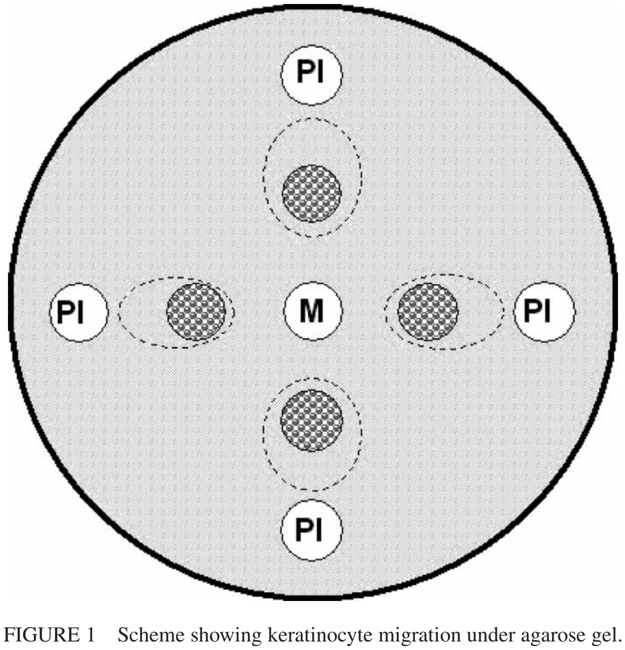

FIGURE 1 Scheme showing keratinocyte migration under agarose gel. Human epidermal keratinocytes were dispensed into the middle wells (shown as pitted dark gray circles) and were allowed to migrate under agarose gel for 24–48 h toward plasmin (Pl; chemotaxis) or medium (M; spontaneous migration). Dashed lines represent typical (oval) pattern of cell migration.

the distance of migration induced by plasmin and that seen by spontaneous migration.

### *Microchemotaxis*

Keratinocyte migration in serum free conditions was studied using a 48-well microchemotaxis chamber (Neuro Probe, Gaithersburg, MD, USA) with a 12 μm pore size PVP-free uncoated membrane (Osmonics, Livermore, CA, USA). These experiments were carried out on HaCaT cells. For migration studies the cell number was adjusted to $1 \times 10^6$/ml in binding medium (RPMI 1640 supplemented with 1% BSA and 25 mM Hepes, both from GibcoBRL). The bottom chamber was filled with the chemoattractant or with medium for purposes of control. Cells were loaded into the upper chamber and were incubated at 37°C, 5% $CO_2$ for 45 min. The plasmin inhibitor Serpin was added to the bottom wells, or in separate experiments cells were preincubated with or without Serpin for 15 min at 37° C, 5% $CO_2$. Chemokinesis was analyzed by adding plasmin together with HaCaT cells into the upper chamber, while the lower chamber contained binding medium. At the end of the migration period, the membrane was removed, and the top surface was wiped to remove non-migrating cells. The membrane was fixed in methanol and stained with hematoxylin eosin. The chemotaxis was evaluated by counting the stained migrating cells at the bottom of the membrane in four representative fields by light microscopy at low magnification (200 × ). Each culture combination was performed in triplicate and each experimental protocol was performed in three separate experiments.

### Organisms and Culture Conditions

A clinical isolate of *Candida albicans* strain was used throughout these studies. Stock cultures were maintained by serial passage on Sabouraud dextrose agar plates (Difco, Sparks, MD, USA) at room temperature. For use, yeast cells were grown for 18 h, washed three times in PBS, and adjusted to the appropriate cell density in R10 without amphotericin B.

### Assay for Candida Growth

Ten thousand keratinocytes were allowed to adhere to the bottom wells of 96 well plates for 8–9 h, washed with an antimycotic free medium and were infected with one thousand *Candida albicans* yeast cells resulting in a 10:1 effector:target ratio. Plasmin (25 U/l) and its inhibitor $\alpha_2$-antiplasmin (25 U/l) was added together with Candida and the culture was further incubated at 37°C, 5% $CO_2$ for 12–14 h. During this culture period Candida yeast cells transformed to a highly adherent hyphae form with intense metabolic activity. Candida growth (i.e. yeast–hyphae transformation) was monitored by the MTT assay (described below) utilizing dehydrogenase activity of live cells. Adhering keratinocytes were removed by adding 0.1% Triton X-100 (TTX, Sigma) for 10 min into the wells. TTX treatment exerted no detectable effect on Candida adhesion or dehydrogenase activity.

### MTT Assay

Quantitation of Candida growth by tetrazolium-based colorimetric assay was performed as described by Levitz and Diamond (1985). Microtiter wells containing adherent cells were incubated for 12–14 h under the described experimental conditions, washed twice with PBS and 5 μg/well of MTT 3-[4,5-Dimethylthiazol-2-yl]-2,5-diphenyltetrazolium bromide, (Sigma) diluted in RPMI 1640 without phenol red (GibcoBRL) was added for 4 h at 37°C. Conversion of MTT to insoluble MTT-formazan by cell-derived dehydrogenases was quantitated after solubilization of MTT-formazan with 10% SDS (sodium dodecyl sulphate; Sigma) and warm acid alcohol. The change in O.D. at 595 nm was determined using a microELISA reader.

### Assay for Proliferation

Ten thousand cells were incubated in individual wells of 96 well plates at 37°C, 5% $CO_2$ for 24 and 48 h in the presence of 25 U/l plasmin; 25 U/l plasmin + 25 U/l $\alpha_2$-antiplasmin (Serpin) or in medium as control. $^3$H-Thymidine uptake was determined following the addition of 2 μCi (74 KBq) $^3$H-Thymidine (Amersham Life Sciences, Braunschweig, Germany) to each well for 6 h. Cells were harvested after detachment with 1.25% Trypsin (Sigma). Cultures were performed in triplicate and the assay was repeated three times.

### Statistical Analysis

The results are presented as mean ± SD. For each analysis, the comparison of three or more groups of data was performed using the Kolmogorov–Smirnov test to evaluate normality, and the Levene Median test was performed to evaluate equal variance of the data. If the data was found to satisfy the tests for normality and equal variance, an analysis of variance was performed. If not, the Kruskal–Wallis non-parametric ANOVA was performed. For those analyses comparing the mean of one group with that of another group, we tested for normality as above. If the data passed the normality test, we used the two-tailed unpaired *t*-test. The level of significance was set at 0.05 in all cases.

### RESULTS

### Keratinocyte Migration in the Presence of Serum

Migration of freshly isolated HEK was studied utilizing the agarose gel assay which permits the examination of both spontaneous and chemotactic migration of aliquots of the same group of cells in the presence of human

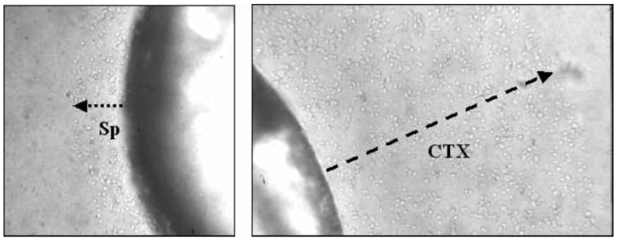

FIGURE 2 Migrating keratinocytes under agarose gel. Microscopic pictures show spontaneous (Sp, toward medium; left panel) and chemotactic (CTX, toward plasmin; right panel) migration of human epidermal keratinocyte. Cell migration was quantified microscopically by the measurement of the distance between the leading edge of migrating cells and the well border, as shown by dashed arrows.

serum. Plasmin at the concentration of 25 U/l significantly enhanced the migration activity of keratinocytes compared to spontaneous migration (Fig. 2). As seen, keratinocytes appear to migrate on plastic surfaces under the agarose gel toward both medium (spontaneous migration, Sp) and plasmin (chemotaxis, CTX). Evaluation of cell migration by the measurement of the distance that keratinocytes traveled during the incubation period revealed a mean value of a 1.6-fold increase in response to plasmin as compared to medium alone (migration index = 2.6). As seen in Fig. 3, addition of the plasmin inhibitor $\alpha_2$-antiplasmin (Serpin) at a concentration of 25 U/l completely blocked plasmin effect. Similar results were obtained when Serpin was added either to the cell containing wells (top bar) or together with plasmin (second bar from the top). Serpin at the concentration of 25 U/l, however, did not appear to influence the spontaneous migration of keratinocytes (third bar from the top).

### Keratinocyte Migration in Serum Free Conditions

Plasmin induced keratinocyte migration was further analyzed in serum free conditions. In these experiments the immortalized human epidermal keratinocyte cell line, HaCaT was used because of its high adhesion capacity. Plasmin induced a dose-dependent increase in HaCaT cell migration with the maximum at 25 U/l concentration

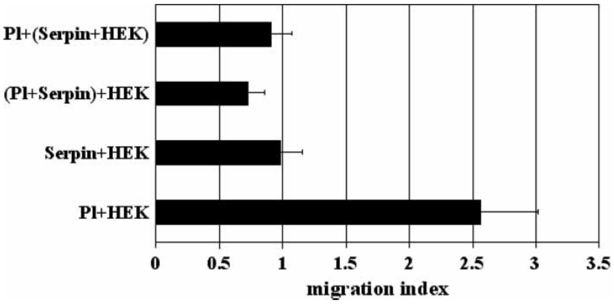

FIGURE 3 Evaluation of keratinocyte migration under agarose gel. Plasmin at the concentration of 25 U/l induced enhanced migration of human epidermal keratinocytes. Migration index was expressed as the ratio of chemotactic and spontaneous migration distance. The plasmin inhibitor Serpin at the concentration of 25 U/l completely blocked plasmin effect. Data represent the mean ± SD of five experiments.

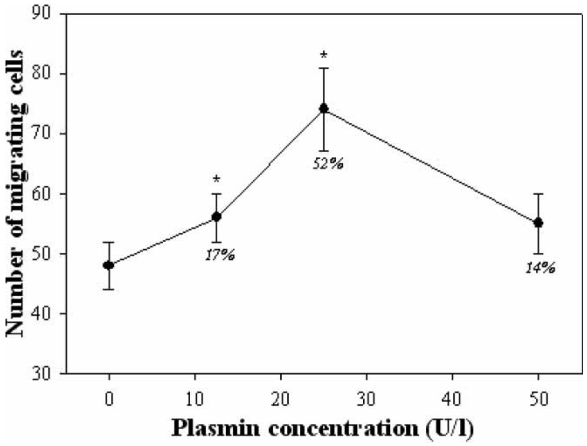

FIGURE 4 Chemotaxis of HaCaT keratinocytes toward plasmin in serum free milieu. The chemotactic responsiveness of the HaCaT cell line was determined by utilizing the microchemotaxis assay. Migration toward the designated concentrations of plasmin was terminated after 45 min incubation at 37°C, and the migrating cells were then stained and counted. Each data point represents the mean ± SD of three experiments in triplicate. Percent increase in the number of migrating cells compared to migration toward medium is also shown. $*P < 0.05$.

(Fig. 4). Further increase in plasmin concentration decreased the chemotactic responsiveness resulting in a bell shape dose-response curve, characteristic for "classical" chemotaxis. In order to clarify whether the enhanced cell migration toward plasmin was due to chemotaxis or it was associated with cell activation, experiments were carried out to analyze plasmin chemokinesis. In these experiments HaCaT cells were loaded together with plasmin into the upper wells of a microchemotaxis chamber, while the bottom wells were filled with binding medium. In such culture conditions, plasmin induced no significant change in cell migration suggesting insignificant chemokinetic effect on HaCaT cells at the dose range examined (Fig. 5).

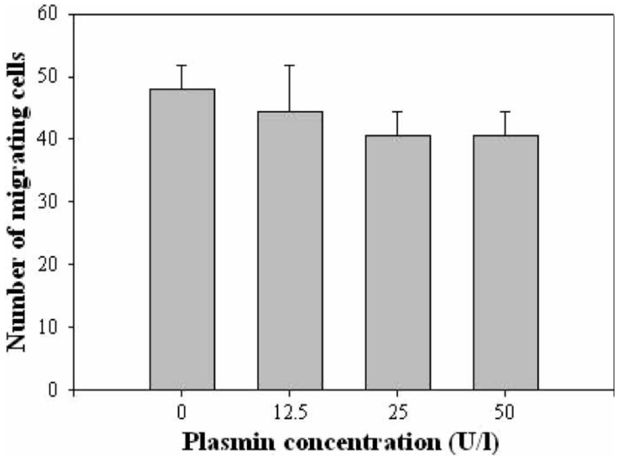

FIGURE 5 Analysis of chemokinetic effect of plasmin on HaCaT keratinocytes. Plasmin chemokinesis was determined by the microchemotaxis assay. Cell suspensions were prepared with the designated concentrations of plasmin, and were allowed to migrate toward medium for 45 min as described in Fig. 4. Each bar represents the mean ± SD of three experiments each performed in triplicate.

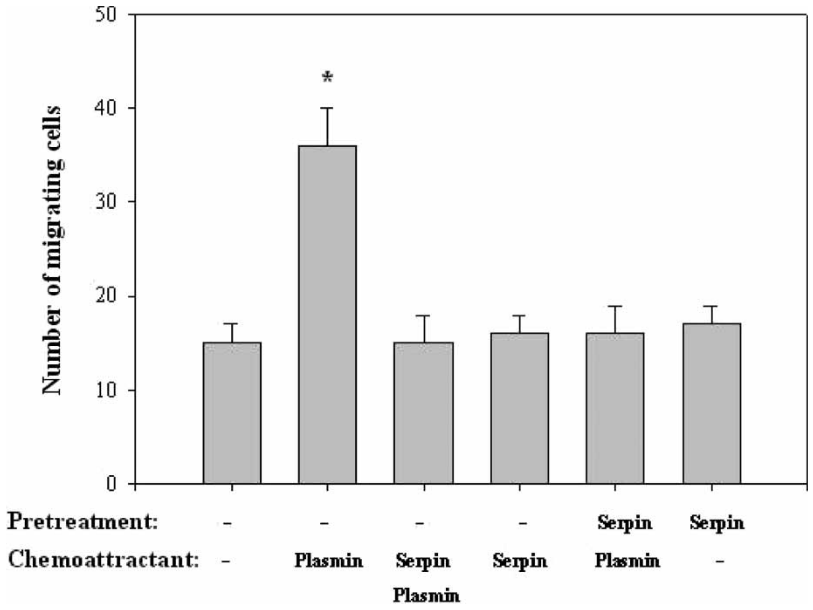

FIGURE 6 Inhibition of plasmin induced keratinocyte migration by $\alpha_2$-antiplasmin (Serpin). HaCaT cells were pre-incubated either in the presence or absence of 25 U/l Serpin for 15 min at 37°C and the chemotactic migration toward the designated combination of plasmin and/or Serpin was determined as described in Fig. 4. Each bar represents the mean ± SD of three experiments each performed in triplicate. $*P < 0.05$.

Additional experiments with the plasmin inhibitor $\alpha_2$-antiplasmin were carried out to study the specificity of plasmin induced chemotaxis. In these experiments HaCaT cells were pre-incubated in the presence of 25 U/l $\alpha_2$-antiplasmin or in binding medium for 15 min at 37°C and 5% $CO_2$, and the spontaneous and chemotactic migration was determined. Figure 6 shows that the presence of or pretreatment with the plasmin inhibitor completely abolished the plasmin effect. On the other hand, the addition of $\alpha_2$-antiplasmin had no effect on the spontaneous cell migration. Interestingly the 15 min pre-incubation at 37°C somewhat decreased the baseline migration of HaCaT cells presumably due to enhanced adhesion capacity.

### Keratinocyte-mediated Inhibition of Candida Growth

It was hypothesized that migrating keratinocytes ingest and process debris during trafficking. In order to study the effect of plasmin on this process, HEKs were co-cultured with live *Candida albicans* and the keratinocyte-mediated inhibition of fungal growth was determined by the MTT colorimetric assay. The incubation of Candida with freshly isolated HEKs at the ratio of 10:1 resulted in a marked decrease in fungal growth (Fig. 7). Plasmin at the concentration of 25 U/l further increased keratinocyte-mediated inhibition of Candida growth, which was completely reversed by 25 U/l Serpin. Serpin alone exerted no modulatory effect. In addition, Candida growth was not altered in the presence of plasmin or Serpin (not shown).

### Keratinocyte Proliferation

Experimental data suggests that there is an inverse regulation of keratinocyte migration and proliferation during wound healing (Garlick and Taichman, 1994). We were therefore interested in determining whether the plasmin induced increase in keratinocyte migration was also associated with suppressed cell proliferation activity. These experiments were carried out using the HaCaT cell line, which require no external growth factors for proliferation, which could potentially be influenced by the protease activity of plasmin. The effect of plasmin on HaCaT proliferation was thus examined by measuring the uptake of $^3$H-Thymidine (Fig. 8). Keratinocytes from pre-sheet cultures were incubated for 24 and 48 h in the presence of plasmin, its inhibitor Serpin, or in the medium as control. Plasmin at the concentration of 25 U/l induced 43 and 17% inhibition of $^3$H-Thymidine incorporation after 24 and 48 h incubation respectively, which was reversed by 25 U/l Serpin. Serpin on the other hand, had no effect on Thymidine uptake.

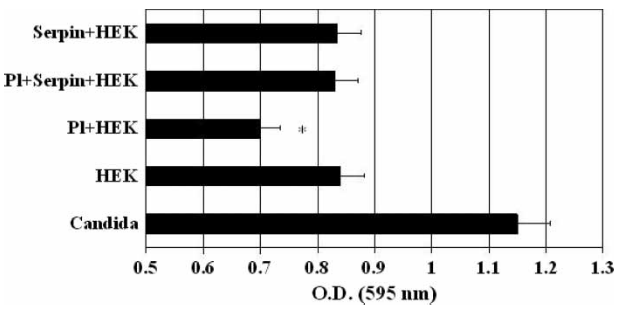

FIGURE 7 The effect of plasmin on HEK-mediated inhibition of the growth of Candida. Human epidermal keratinocytes were incubated in the presence or absence of 25 U/l plasmin with *Candida albicans* yeast cells at the ratio of 10:1 for 12–14 h. Candida growth was determined by the MTT colorimetric assay. Results shown are the mean ± SD of five experiments. $*P < 0.05$.

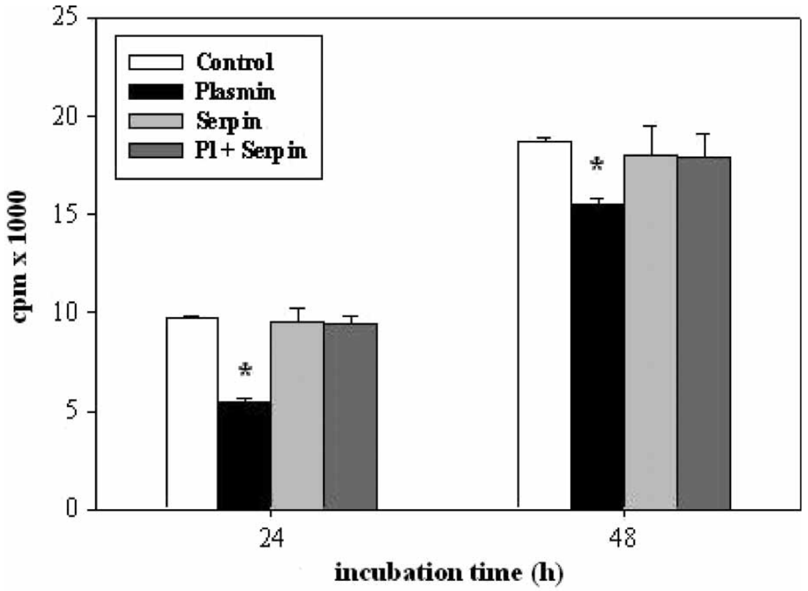

FIGURE 8 The effect of plasmin on HaCaT proliferation. Ten thousand cells were incubated in individual wells of 96 well plates for 24 and 48 h in the presence of 25 U/ml plasmin, 25 U/l Serpin or in the medium as control. $^{3}$H-Thymidine uptake was determined after incubating cells with the isotope for 6 h. Measurements were carried out in triplicate and repeated three times. Results shown are the mean ± SD of the three experiments. $^{*}P < 0.05$.

### DISCUSSION

The plasminogen/plasmin system has been previously shown to modulate cell adhesion, and initiate both a conformational change and cell locomotion (Erickson and Isseroff, 1989; Stephens *et al.*, 1989; Barnathan *et al.*, 1990; Goldberg *et al.*, 1990; Inyang and Tobelem, 1990; Oikarinen *et al.*, 1990; Plow and Miles, 1990; Hart *et al.*, 1991; Clowes *et al.*, 1992; Hoekman *et al.*, 1992; Meissauer *et al.*, 1992; Schmitt *et al.*, 1992; Shea and Beermann, 1992; Sperti *et al.*, 1992; Blasi, 1993; Ciacci *et al.*, 1993; Pepper *et al.*, 1993; Wojta *et al.*, 1993: Damjanovich *et al.*, 1994; Syrovets *et al.*, 1997). Many cell lineages respond to plasmin with increased migration activity. These cell lineages include monocytes, neurons, vascular smooth muscle and endothelial cells. In addition to an increase in normal cell locomotion, plasmin was reported to induce tumor cell invasion (Goldberg *et al.*, 1990; Meissauer *et al.*, 1992; Blasi, 1993; Damjanovich *et al.*, 1994). Keratinocytes have been found to activate the plasminogen/plasmin system (Isseroff and Rifkin, 1983; Hashimoto *et al.*, 1988; McNeill and Jensen, 1990; Reinartz *et al.*, 1993; Reinartz *et al.*, 1994; Reinartz *et al.*, 1995; Reinartz *et al.*, 1996). In the re-epithelialization during wound healing, an intense plasminogen activator activity could be detected on keratinocytes (Grondahl-Hansen *et al.*, 1988; Baird *et al.*, 1990; McNeill and Jensen, 1990; Reinartz *et al.*, 1994; Bechtel *et al.*, 1996). Experimental data suggest that serum from the wound infiltrating blood can activate keratinocyte-derived PA (Chen and Jensen, 1996). PA mediated plasminogen conversion to plasmin leads to pericellular proteolysis by its serine protease activity. However, indirect evidences also suggest a number of non-proteolytic effects of plasmin on keratinocytes (Burge *et al.*, 1992).

Our results show that plasmin at a concentration of 25 U/l induced a 1.6-fold increase in chemotactic migration of HEKs in a serum-containing milieu, which was completely reversed by the plasmin inhibitor Serpin. Although plasmin has been shown previously to induce chemotaxis of several human cell lineages, according to our knowledge this is the first report of the effect of plasmin on HEKs. In these experiments we applied the agarose gel assay to study keratinocyte migration on the plastic surface of tissue culture plates (Petri dish) under agarose gel, containing 10% human AB serum (Nelson *et al.*, 1975). In such conditions the protease activity of plasmin may contribute to the enhanced locomotion of cells, although the chemotactic concentration (7.8 μg/ml) was far below the precursor plasminogen normal blood level (200 μg/ml) (Leipnitz *et al.*, 1988; Tait *et al.*, 1992). In order to study the non-proteolytic effect of plasmin on the chemotactic migration of keratinocytes we carried out experiments in the absence of serum using the microchemotaxis method. In these experiments the highly adherent and rapidly growing immortalized human keratinocyte cell line, HaCaT cells, were used which allowed for repeated analysis of essentially identical cells. We found that plasmin induced a dose-dependent chemotaxis of HaCaT cells with the maximum effect (52% increase) at a concentration of 25 U/l. Dose-response analysis showed that plasmin, similar to "classical" chemokines induced a bi-phasic effect (Szabo *et al.*, 2001; Szabo *et al.*, 2002): i.e. increasing plasmin concentration over the maximum effect led to a decrease in the chemotactic responsiveness resulting in a bell-shaped curve. Chemotactic migration of HaCaT keratinocytes toward plasmin was reversed by the plasmin inhibitor $\alpha_2$-antiplasmin, which on the other hand, induced no detectable effect on spontaneous cell locomotion.

Enhanced cell migration *in vitro* is associated with certain cell activation processes leading to higher mobility of cells, designated chemokinesis. Our results, however, revealed no chemokinetic effect of plasmin on HaCaT keratinocytes at the concentration range examined. These data provide strong evidence that plasmin-induced keratinocyte migration indeed represents chemotaxis.

It is believed that migrating keratinocytes during re-epithelialization *in vivo* not only use the wound bed for trafficking, but also "clean" their way by ingesting and processing debris. We attempted to model this process by co-culturing live *Candida albicans* yeast cells with HEKs. In appropriate culture conditions Candida yeast cells transform to a very adherent and highly resistant hypha form with intense metabolism. Based on their metabolic activity, the growth rate and viability could be monitored by the MTT assay utilizing the intracellular dehydrogenases in live cells (Levitz and Diamond, 1985). The advantage of using Candida for these experiments was their relative resistance to proteolytic digestion (Csato *et al.*, 1986) including the protease activity of plasmin. Culturing Candida in the presence of plasmin indeed resulted in no detectable growth inhibition. HEKs, however, markedly decreased Candida growth. HEKs in the presence of 25 U/l plasmin further reduced the growth rate of Candida, an effect which was inhibited by Serpin. The precise mechanism by which keratinocytes control Candida growth is somewhat controversial. Csato *et al.* (1986) suggest phagocytic-killing of Candida yeast by keratinocytes, other reports claim extracellular killing of fungi (Szolnoky *et al.*, 2001; Pivarcsi *et al.*, 2003). Nonetheless, our results show that plasmin-induced chemotaxis is accompanied by enhanced "cleaning" activity of epidermal keratinocytes, presumably contributing to facilitated migration of cells on wound bed during re-epithelialization.

Reconstitution of epithelial integrity following skin injury has been shown to start with re-epithelialization, the process known to be responsible for the formation of the keratinocyte monolayer on the wound bed (Laplante *et al.*, 2001). It is widely accepted that migration of keratinocytes on the wound bed is continuously supplied by dividing cells at the wound border (Garlick and Taichman, 1994). It is believed that keratinocyte migration and division during re-epithelialization is inversely regulated, i.e. stimulation of keratinocyte migration leads to inhibition of cell proliferation, and locomotion of dividing keratinocytes will be blocked. Tumor growth factor-beta has been reported to induce a similar regulation of intestinal epithelial cells with strong inhibition of proliferation while stimulating cell migration (Ciacci *et al.*, 1993). We were interested whether the plasmin effect on epidermal keratinocytes with enhanced chemotactic migration would follow the above regulatory pattern. In order to study this question, experiments were conducted on HaCaT keratinocytes because they require no external growth factors for proliferation that the plasmin protease activity might interfere with. Spontaneous proliferation of HaCaT keratinocytes incubated in the presence of 25 U/ml plasmin for 24 or 48 h was indeed reduced by 43 and 17%, respectively as determined by $^{3}$H-Thymidine uptake. The inhibitory effect of plasmin was also neutralized by Serpin, which on the other hand, induced no detectable effect on HaCaT proliferation.

In conclusion our results suggest an important modulatory role of plasmin in the early phase of wound healing. Serine protease activity leads to pericellular proteolysis and the consequent mobilization of cells. Moreover, plasmin may induce functional changes of keratinocytes including chemotactic migration with enhanced phagocytic-killing activity, and suppressed cell proliferation, resulting in facilitating the re-epithelialization processes and thereby stimulate wound healing.

### References

Ando, Y. and Jensen, P.J. (1996) "Protein kinase C mediates up-regulation of urokinase and its receptor in the migrating keratinocytes of wounded cultures, but urokinase is not required for movement across a substratum *in vitro*", *J. Cell. Physiol.* **167**, 500–511.

Baird, J., Lazarus, G.S., Belin, D., *et al.* (1990) "mRNA for tissue-type plasminogen activator is present in lesional epidermis from patients with psoriasis, pemphigus, or bullous pemphigoid, but is not detected in normal epidermis", *J. Investig. Dermatol.* **95**, 548–552.

Barnathan, E.S., Kuo, A., Rosenfeld, L., *et al.* (1990) "Interaction of single-chain urokinase-type plasminogen activator with human endothelial cells", *J. Biol. Chem.* **265**, 2865–2872.

Bechtel, M.J., Reinartz, J., Rox, J.M., Inndorf, S., Schaefer, B.M. and Kramer, M.D. (1996) "Upregulation of cell-surface-associated plasminogen activation in cultured keratinocytes by interleukin-1 beta and tumor necrosis factor-alpha", *Exp. Cell Res.* **223**, 395–404.

Blasi, F. (1993) "Urokinase and urokinase receptor: a paracrine/autocrine system regulating cell migration and invasiveness", *Bioessays* **15**, 105–111.

Boxman, I.L., Quax, P.H., Lowik, C.W., Papapoulos, S.E., Verheijen, J. and Ponec, M. (1995) "Differential regulation of plasminogen activation in normal keratinocytes and SCC-4 cells by fibroblasts", *J. Investig. Dermatol.* **104**, 374–378.

Burge, S.M., Marshall, J.M. and Cederholm-Williams, S.A. (1992) "Plasminogen binding sites in normal human skin", *Br. J. Dermatol.* **126**, 35–41.

Chen, C.S. and Jensen, P.J. (1996) "Serum is a potent stimulator of keratinocyte tissue plasminogen activator expression", *J. Investig. Dermatol.* **106**, 238–242.

Ciacci, C., Lind, S.E. and Podolsky, D.K. (1993) "Transforming growth factor beta regulation of migration in wounded rat intestinal epithelial monolayers", *Gastroenterology* **105**, 93–101.

Clowes, A.W., Clowes, M.M., Kirkman, T.R., Jackson, C.L., Au, Y.P. and Kenagy, R. (1992) "Heparin inhibits the expression of tissue-type plasminogen activator by smooth muscle cells in injured rat carotid artery", *Circ. Res.* **70**, 1128–1136.

Csato, M., Bozoki, B., Hunyadi, J. and Dobozy, A. (1986) "*Candida albicans* phagocytosis by separated human epidermis cells", *Arch. Dermatol. Res.* **279**, 137–139.

Damjanovich, L., Turzo, C. and Adany, R. (1994) "Factors involved in the plasminogen activation system in human breast tumours", *Thromb. Haemost.* **71**, 684–691.

Del Rosso, M., Fibbi, G., Dini, G., Grappone, C., *et al.* (1990) "Role of specific membrane receptors in urokinase-dependent migration of human keratinocytes", *J. Investig. Dermatol.* **94**, 310–316.

Erickson, C.A. and Isseroff, R.R. (1989) "Plasminogen activator activity is associated with neural crest cell motility in tissue culture", *J. Exp. Zool.* **251**, 123–133.

Garlick, J.A. and Taichman, L.B. (1994) "Fate of human keratinocytes during reepithelialization in an organotypic culture model", *Lab. Investig.* **70**, 916–924.

Geer, D.J. and Andreadis, S.T. (2003) "A novel role of fibrin in epidermal healing: plasminogen-mediated migration and selective detachment of differentiated keratinocytes", *J. Investig. Dermatol.* **121**, 1210–1216.

Goldberg, G.I., Frisch, S.M., He, C., Wilhelm, S.M., Reich, R. and Collier, I.E. (1990) "Secreted proteases. Regulation of their activity and their possible role in metastasis", *Ann. N Y Acad. Sci.* **580**, 375–384.

Grondahl-Hansen, J., Lund, L.R., Ralfkiaer, E., Ottevanger, V. and Dano, K. (1988) "Urokinase- and tissue-type plasminogen activators in keratinocytes during wound reepithelialization *in vivo*", *J. Investig. Dermatol.* **90**, 790–795.

Hall, S.W., Humphries, J.E. and Gonias, S.L. (1991) "Inhibition of cell surface receptor-bound plasmin by alpha 2-antiplasmin and alpha 2-macroglobulin", *J. Biol. Chem.* **266**, 12329–12336.

Hart, P.H., Vitti, G.F., Burgess, D.R., Whitty, G.A., Royston, K. and Hamilton, J.A. (1991) "Activation of human monocytes by granulocyte-macrophage colony-stimulating factor: increased urokinase-type plasminogen activator activity", *Blood* **77**, 841–848.

Hashimoto, K., Prystowsky, J.H., Baird, J., Lazarus, G.S. and Jensen, P.J. (1988) "Keratinocyte urokinase-type plasminogen activator is secreted as a single chain precursor", *J. Investig. Dermatol.* **90**, 823–828.

Hoekman, K., Lowik, C.W., van de Ruit, M., Bijvoet, O.L., Verheijen, J.H. and Papapoulos, S.E. (1992) "The effect of tissue type plasminogen activator (tPA) on osteoclastic resorption in embryonic mouse long bone explants: a possible role for the growth factor domain of tPA", *Bone Miner.* **17**, 1–13.

Inyang, A.L. and Tobelem, G. (1990) "Tissue-plasminogen activator stimulates endothelial cell migration in wound assays", *Biochem. Biophys. Res. Commun.* **171**, 1326–1332.

Isseroff, R.R. and Rifkin, D.B. (1983) "Plasminogen is present in the basal layer of the epidermis", *J. Investig. Dermatol.* **80**, 297–299.

Laplante, A.F., Germain, L., Auger, F.A. and Moulin, V. (2001) "Mechanisms of wound reepithelialization: hints from a tissue-engineered reconstructed skin to long-standing questions", *FASEB J.* **15**, 2377–2389.

Leipnitz, G., Miyashita, C., Heiden, M., von Blohn, G., Kohler, M. and Wenzel, E. (1988) "Reference values and variability of plasminogen in healthy blood donors and its relation to parameters of the fibrinolytic system", *Haemostasis* **18** (Suppl.1), 61–68.

Levitz, S.M. and Diamond, R.D. (1985) "A rapid colorimetric assay of fungal viability with the tetrazolium salt MTT", *J. Infect. Dis.* **152**, 938–945.

Lijnen, H.R. (1996) "Pathophysiology of the plasminogen/plasmin system", *Int. J. Clin. Lab. Res.* **6**, 1–6.

McNeill, H. and Jensen, P.J. (1990) "A high-affinity receptor for urokinase plasminogen activator on human keratinocytes: characterization and potential modulation during migration", *Cell Regul.* **1**, 843–852.

Meissauer, A., Kramer, M.D., Schirrmacher, V. and Brunner, G. (1992) "Generation of cell surface-bound plasmin by cell-associated urokinase-type or secreted tissue-type plasminogen activator: a key event in melanoma cell invasiveness *in vitro*", *Exp. Cell Res.* **199**, 179–190.

Nelson, R.D., Quie, P.G. and Simmons, R.L. (1975) "Chemotaxis under agarose: a new and simple method for measuring chemotaxis and spontaneous migration of human polymorphonuclear leukocytes and monocytes", *J. Immunol.* **115**, 1650–1656.

Oikarinen, A., Hoyhtya, M. and Jarvinen, M. (1990) "Dexamethasone-induced plasminogen activator inhibitor: characterization, purification, and preparation of monoclonal antibodies", *Arch. Dermatol. Res.* **282**, 153–158.

Pepper, M.S., Sappino, A.P., Stocklin, R., Montesano, R., Orci, L. and Vassalli, J.D. (1993) "Upregulation of urokinase receptor expression on migrating endothelial cells", *J. Cell Biol.* **122**, 673–684.

Pivarcsi, A., Bodai, L., Rethi, B., *et al.* (2003) "Expression and function of Toll-like receptors 2 and 4 in human keratinocytes", *Int. Immunol.* **6**, 721–730.

Plow, E.F. and Miles, L.A. (1990) "Plasminogen receptors in the mediation of pericellular proteolysis", *Cell Differ. Dev.* **32**, 293–298.

Reinartz, J., Batrla, R., Boukamp, P., Fusenig, N. and Kramer, M.D. (1993) "Binding and activation of plasminogen at the surface of human keratinocytes", *Exp. Cell Res.* **208**, 197–208.

Reinartz, J., Link, J., Todd, R.F. and Kramer, M.D. (1994) "The receptor for urokinase-type plasminogen activator of a human keratinocyte line (HaCaT)", *Exp. Cell Res.* **214**, 486–498.

Reinartz, J., Schaefer, B., Batrla, R., Klein, C.E. and Kramer, M.D. (1995) "Plasmin abrogates alpha v beta 5-mediated adhesion of a human keratinocyte cell line (HaCaT) to vitronectin", *Exp. Cell Res.* **220**, 274–282.

Reinartz, J., Schaefer, B., Bechtel, M.J. and Kramer, M.D. (1996) "Plasminogen activator inhibitor type-2 (PAI-2) in human keratinocytes regulates pericellular urokinase-type plasminogen activator", *Exp. Cell Res.* **223**, 91–101.

Rheinwald, J.G. and Green, H. (1975) "Serial cultivation of strains of human epidermal keratinocytes: the formation of keratinizing colonies from single cells", *Cell* **6**, 331–343.

Rox, J.M., Reinartz, J. and Kramer, M.D. (1996) "Interleukin-1 beta upregulates tissue-type plasminogen activator in a keratinocyte cell line (HaCaT)", *Arch. Dermatol. Res.* **288**, 554–558.

Schmitt, M., Janicke, F., Moniwa, N., Chucholowski, N., Pache, L. and Graeff, H. (1992) "Tumor-associated urokinase-type plasminogen activator: biological and clinical significance", *Biol. Chem. Hoppe-Seyler.* **373**, 611–622.

Shea, T.B. and Beermann, M.L. (1992) "Regulation of neuronal migration and neuritogenesis by distinct surface proteases. Relative contribution of plasmin and a thrombin-like protease", *FEBS Lett.* **307**, 190–194.

Sperti, G., van Leeuwen, R.T., Quax, P.H., Maseri, A. and Kluft, C. (1992) "Cultured rat aortic vascular smooth muscle cells digest naturally produced extracellular matrix. Involvement of plasminogen-dependent and plasminogen-independent pathways", *Circ. Res.* **71**, 385–392.

Stephens, R.W., Pollanen, J., Tapiovaara, H., *et al.* (1989) "Activation of pro-urokinase and plasminogen on human sarcoma cells: a proteolytic system with surface-bound reactants", *J. Cell Biol.* **108**, 1987–1995.

Syrovets, T., Tippler, B., Rieks, M. and Simmet, T. (1997) "Plasmin is a potent and specific chemoattractant for human peripheral monocytes acting via a cyclic guanosine monophosphate-dependent pathway", *Blood* **89**, 4574–4583.

Szabo, I., Wetzel, M.A. and Rogers, T.J. (2001) "Cell-density-regulated chemotactic responsiveness of keratinocytes *in vitro*", *J. Investig. Dermatol.* **117**, 1083–1090.

Szabo, I., Chen, X.H., Xin, L., Adler, M.W., Howard, O.M., Oppenheim, J.J. and Rogers, T.J. (2002) "Heterologous desensitization of opioid receptors by chemokines inhibits chemotaxis and enhances the perception of pain", *Proc. Natl Acad. Sci. USA* **99**, 10276–10281.

Szolnoky, G., Bata-Csorgo, Z., Kenderessy, A.S., *et al.* (2001) "A mannose-binding receptor is expressed on human keratinocytes and mediates killing of *Candida albicans*", *J. Investig. Dermatol.* **117**, 205–213.

Tait, R.C., Walker, I.D., Conkie, J.A., *et al.* (1992) "Plasminogen levels in healthy volunteers—influence of age, sex, smoking and oral contraceptives", *Thromb. Haemost.* **68**, 506–510.

Venning, V.A., Wojnarowska, F. and Cederholm-Williams, S. (1993) "An immunohistochemical study of the distribution of plasminogen and plasminogen activators in bullous pemphigoid", *Clin. Exp. Dermatol.* **18**, 119–123.

Wojta, J., Gallicchio, M., Zoellner, H., Filonzi, E.L., Hamilton, J.A. and McGrath, K. (1993) "Interleukin-4 stimulates expression of urokinase-type-plasminogen activator in cultured human foreskin microvascular endothelial cells", *Blood* **81**, 3285–3292.

Xu, S., Savage, P., Burton, J.L., Sansom, J. and Holly, J.M. (1997) "Proteolysis of insulin-like growth factor-binding protein-3 by human skin keratinocytes in culture in comparison to that in skin interstitial fluid: the role and regulation of components of the plasmin system", *J. Clin. Endocrinol. Metab.* **82**, 1863–1868.

